# Synthesis of Water-Soluble Antimony Sulfide Quantum Dots and Their Photoelectric Properties

**DOI:** 10.1186/s11671-017-2421-1

**Published:** 2018-01-15

**Authors:** Jiang Zhu, Xuelian Yan, Jiang Cheng

**Affiliations:** 1 Co-Innovation Center for Micro/Nano Optoelectronic Materials and Devices, College of Materials and Chemical Engineering, Chongqing University of Arts and Sciences, No. 319, Honghe Road, Yongchuan District, Chongqing, 402160 People’s Republic of China; 2 State Key Laboratory of Advanced Chemical Power Sources, No. 705, Zhonghuabei Road, Honghuagang District, Zunyi, 563003 Guizhou Province People’s Republic of China

**Keywords:** Sb_2_S_3_, Water-soluble quantum dot, Near-infrared emission, Photovoltaic material

## Abstract

Antimony sulfide (Sb_2_S_3_) has been applied in photoelectric devices for a long time. However, there was lack of information about Sb_2_S_3_ quantum dots (QDs) because of the synthesis difficulties. To fill this vacancy, water-soluble Sb_2_S_3_ QDs were prepared by hot injection using hexadecyltrimethylammonium bromide (CTAB) and sodium dodecyl sulfate (SDS) mixture as anionic-cationic surfactant, alkanol amide (DEA) as stabilizer, and ethylenediaminetetraacetic acid (EDTA) as dispersant. Photoelectric properties including absorbing and emission were characterized by UV-Vis-IR spectrophotometer and photoluminescence (PL) spectroscopic technique. An intensive PL emission at 880 nm was found, indicating Sb_2_S_3_ QDs have good prospects in near-infrared LED and near-infrared laser application. Sb_2_S_3_ QD thin films were prepared by self-assembly growth and then annealed in argon or selenium vapor. Their band gaps (*E*_*g*_s) were calculated according to transmittance spectra. The *E*_*g*_ of Sb_2_S_3_ QD thin film has been found to be tunable from 1.82 to 1.09 eV via annealing or selenylation, demonstrating the good prospects in photovoltaic application.

## Background

Quantum dots (QDs) have received a great deal of attention over the past decade owing to their manipulated photoelectric properties and superior solution processibility for device engineering [1–3]. Typically, lead compound QDs such as PbS and lead halide perovskites have recently emerged as promising candidate materials in photoelectric applications such as photovoltaics, OLEDs, lasing, and photodetectors due to their simple synthesis and satisfactory performance [4–6]. Besides, a range of semiconductor QDs, such as CdS, CdSe, ZnS, ZnSe, HgTe, CuInSe_2_, CuInS_2_, and CdHgTe, and base device have been reported everywhere.

Sb_2_S_3_ has been known as the commonest antimony sulfide, which is a promising semiconductor material for photoelectric semiconductor manufacturing [7, 8]. It has a moderate band gap approximately 1.7–1.8 eV in crystalline form (stibnite). Curiously, the band gap is tunable at the range of 1.1–1.8 eV when sulfur is partly replaced by selenium [9]. Naturally, Sb_2_S_3_ is a multifunction material that could be used as an absorber or a sensitizer for photovoltaic device, photochemical catalysis, and photodetector. Besides, Sb and S are comparatively abundant, low-cost, and low-toxicity elements, making it potential for large-scale application. Antimony sulfide has a unique processibility. They can be vacuum-evaporated at a low temperature (~ 400 °C) or solution-processed using various materials. Sb_2_S_3_ was usually applied in sensitized solar cells. Using a thioacetamide-treated Sb_2_S_3_ sensitizer deposited by chemical solution deposition (CBD), a sensitized hybrid solar cell with a power conversion efficiency (PCE) of 7.5% was realized [10]. Recently, solution-processed planar heterojunction solar cells with a simple structure achieved a very satisficing PCE of 4.3%, in which an Sb_2_S_3_ film was prepared by conventional spin-cast technique with a precursor containing Sb_2_O_3_, CS_2_, and n-butylamine [7]. Nanostructure Sb_2_S_3_ synthesized by solution method was wildly applied for high-performance photodetectors [11–13]. Sb_2_S_3_ NW-based photodetectors exhibited a good photo-response in a wide spectral range from 300 to 800 nm. Especially at 638 nm, they showed optimal values with a high current ON/OFF ratio about 210, a spectral responsivity of 1152 A/W, a detectivity of 2 × 10^13^ Jones, and the rise and fall times of about 37 ms [11]. Solution-processed Sb_2_S_3_ nanorod was usually used as an efficient photocatalyst for dye degradation [14] and high-performance sodium-ion batteries [15]. Unfortunately, there was few reported information about Sb_2_S_3_ QDs.

We believe Sb_2_S_3_ zero-dimensional materials must have unusual optical and electrical properties comparing to multidimensional materials because of the quantum confinement effect. To fill this vacancy, the present paper firstly reported the synthesis of water-soluble antimony sulfide QDs using CTAB and SDS mixture as anionic-cationic surfactant, DEA as stabilizer, and EDTA as dispersant under 120 °C oil bath conditions. In order to overcome the interference of hydroxyl, the reaction was conducted in anhydrous 2-methoxyethanol instead of water. These precursors are nontoxic, odorless, and inexpensive compared with conventional additives [16, 17]. Before the substantial application, the structural, optical, and electrical properties were studied herein.

### Experimental

Sb_2_S_3_ QDs were synthesized by rapid hot injection method. In a typical procedure for the preparation, SDS (0.05 mmol, 99.5%), CTAB (0.05 mmol, 99.5%), EDTA (0.2 mmol, 99.5%), and DEA (4 ml, 99.9%) were mixed in the 100-ml anhydrous 2-methoxyethanol and dissolved after 20 min magnetic stirring in 120 °C oil bath. Next, 0.5 mmol thioacetamide (TAA) was dissolved in the hot solution. Then, 2 ml antimony acetate—acetic acid solution (0.25 M)—was injected to the precursor solution with magnetic stirring. Immediately, the solution turned from transparent to bright yellow, indicating the formation of sulfide. The container was then turned into ice bath to terminate reaction. The final product was centrifuged at 15000 rpm for 10 min and then washed with isopropanol repeatedly for at least three times and finally was centrifuged at 6000 rpm for 5 min to remove the coarse particles.

Sb_2_S_3_ QDs were vacuum-dried at room temperature and then tested using a simultaneous thermal analyzer (STA 449 F3, NETZSCH). Crystal structure was characterized by X-ray diffraction (XRD, Bruker D8). Composition measurement was carried out by an energy-dispersive spectrometer (EDS, EDAX Inc.). Sb_2_S_3_ powder (99.99%, Aladdin) was used as standard for the calibration of EDS measurements. Nanoscale information of QDs was characterized by high-resolution transmission electron microscopy (HRTEM; Zeiss Libra200) with selected-area electron diffraction (SAED). The emission spectra were recorded by using photoluminescence spectroscopic technique (PL, Horiba iHR550) with an He–Ne laser (325 nm) as excitation source. Optical transmittance spectra were carried out on QD dispersion and films by using a UV-Vis-IR spectrophotometer (Agilent Cary 5000).

## Results and Discussion

The synthesis of Sb_2_S_3_ QDs is a low-cost, easy operation and repeatable process. The chemical reaction can be described in the following two simple reaction equations.

1 $$ {\mathrm{CH}}_3{\mathrm{CSNH}}_2+2{\mathrm{CH}}_3{\mathrm{OCH}}_2{\mathrm{CH}}_2\mathrm{O}\mathrm{H}\to {\mathrm{CH}}_3\mathrm{C}{\left({\mathrm{CH}}_3{\mathrm{OCH}}_2{\mathrm{CH}}_2\mathrm{O}\right)}_2{\mathrm{NH}}_2+{\mathrm{H}}_2\mathrm{S} $$

2 $$ 2\mathrm{S}\mathrm{b}{\left({\mathrm{CH}}_3\mathrm{COO}\right)}_3+3{\mathrm{H}}_2\mathrm{S}\to {\mathrm{S}\mathrm{b}}_2{\mathrm{S}}_3+6{\mathrm{CH}}_3\mathrm{COO}\mathrm{H} $$

According to the LaMer model [18], separation of nucleation and crystal growth stages is the main requirement for small particle formation with narrow size distributions. At the early stage of this reaction, the solution containing equimolar SDS/CTAB tended to form relatively larger catanionic vesicles rather than mixed micelles [16]. The reaction between S^2−^ and Sb^2+^ took place rapidly, leading to the explosive nucleation. Next, due to the chelation effect, the formation of the metal ions-ETDA complexes reduces the free metal ion concentration [19]. Thus, the grain growth was effectively inhibited, remaining Sb_2_S_3_ QDs in the solution.

**Fig. 1:**
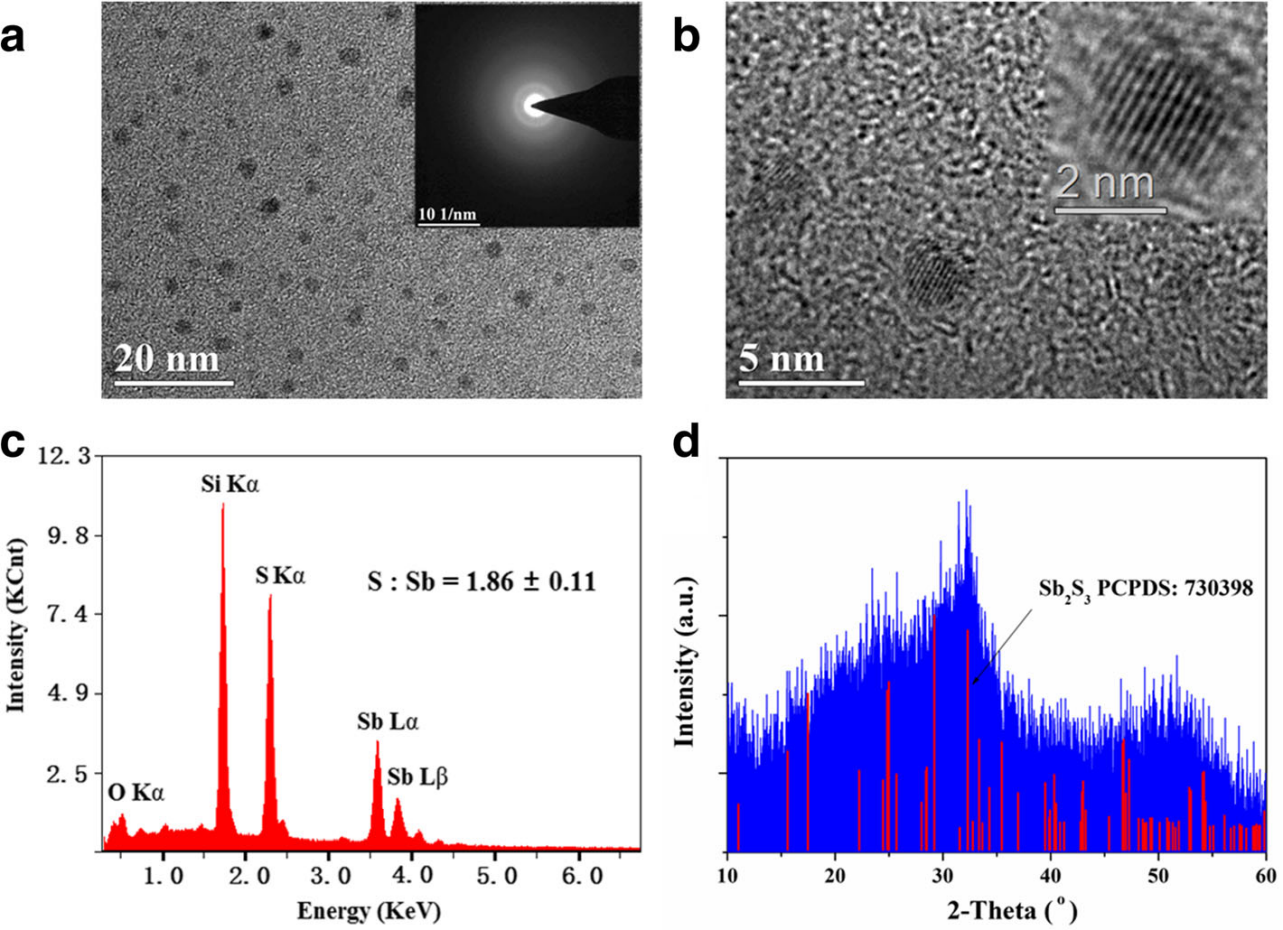
Morphology and structural properties of Sb_2_S_3_ QDs. **a** TEM image and SAED pattern. **b** High-resolution TEM image. **c** EDS analysis and **d** XRD pattern

Effect of temperature and reaction time on the morphologies of QDs has been studied first. We found the shape and size were nearly invariable when the temperature varied from 90 to 120 °C and the reaction time was controlled from 30 s to 15 min. Figure 1a, b shows a TEM image and a high-resolution image of the sample synthesis at 120 °C. The images reveal good monodispersity of QDs with a uniform spherical shape, and the diameters mainly lie in the range of 3 to 5 nm. The high-resolution image shows a clear lattice fringe, revealing each particle is a monocrystalline quantum. SAED exhibits some concentric circles with indistinct boundaries, indicating the synthesized nanomaterial has a low crystallinity. Chemical compositions were analyzed by EDS as shown in Fig. 1c. A quantitative elemental EDS analysis of QDs reveals the average atom ratio (S%:Sb%) is 1.68, indicating the stoichiometric ratio of sulfur element is slightly higher. We deduced that some sulfur was chemisorbed or physically adsorbed on the surface of QDs. Figure 1d shows XRD spectrum of vacuum-dried QDs. Roughly, XRD pattern is matched to orthorhombic Sb_2_S_3_ (JCPDS no. 73-0393), confirming the results of EDS analysis. The indistinct XRD peaks indicate the low crystallinity which is quite agreeable with the SAED pattern.

**Fig. 2:**
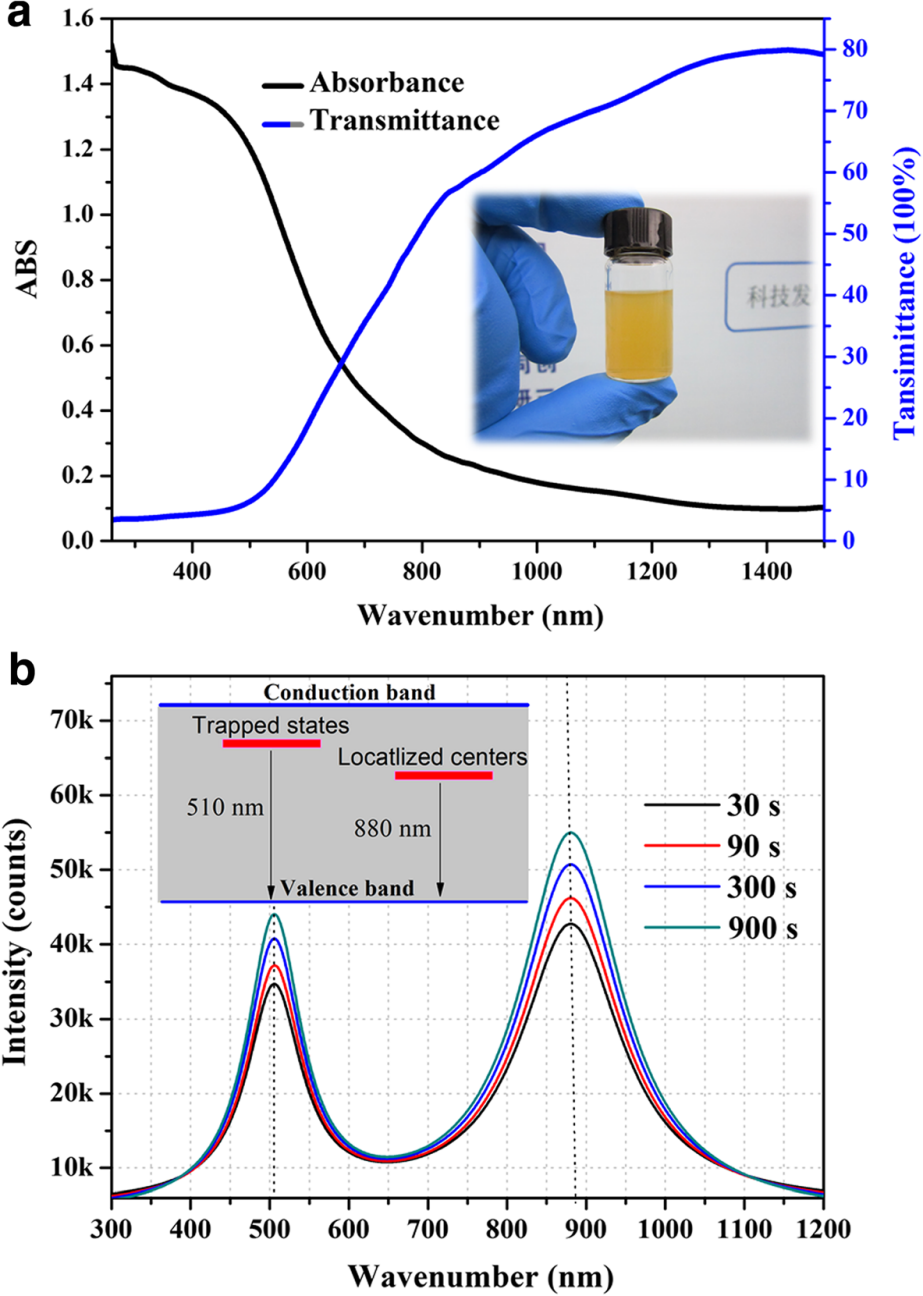
Optical properties of Sb_2_S_3_ QD dispersion. **a** Absorbance and **b** PL spectrum of Sb_2_S_3_ dispersion

Optical absorption of QDs-isopropanol dispersion was measured by Agilent Cary 5000 spectrophotometer. As we see in Fig. 2a, Sb_2_S_3_ QDs dispersion is bright yellow and has a broad absorption at nearly the whole visible range. It shows a nearly complete absorption at short wavelength from 300 to 500 nm and a high transmittance at a near-infrared region. Figure 2b shows the photoluminescence (PL) spectra of dispersion with a concentration of 2 mg/ml where Sb_2_S_3_ was prepared with different reaction times. PL spectra for all Sb_2_S_3_ samples exhibit two distinct emission peaks at around 510 nm (2.43 eV) and 880 nm (1.41 eV), which is significantly different from nanostructured Sb_2_S_3_ prepared by chemical solution deposition (CBD) [20]. According to the previous report, CBD-synthesized Sb_2_S_3_ nanocrystals show a weak band edge emission peaked at around 610 nm (2.03 eV) presumably resulting from excitons and a sulfur vacancy-related strong emission peaked at 717 nm (1.72 eV). For water-soluble Sb_2_S_3_ QDs here, the green emission around 510 nm presumably results from excitons [21, 22], which is well known and widely reported for semiconductor nanocrystals [23], suggesting the quantum size effect (QSE) brings a broader band gap for Sb_2_S_3_ QDs. The near-infrared emission around 880 nm may be attributed to the presence of stoichiometry-related point defects. According to the EDS analysis discussed above, the average atom ratio (S%:Sb%) is 1.68; we deduced sulfur is excessive and the type of point defects here is likely to be antimony vacancies (V^*^_Sb_). Careful observation of curves reveals that the emission peaked at 880 nm of Sb_2_S_3_ QDs prepared with long reaction time exhibits slightly blue shift compared with rapid synthesized QDs. This shift is probable from the slight improvement of stoichiometric ratio. The intensive PL emission and high transmittance at a near-infrared region point that Sb_2_S_3_ QDs have good prospects in the fabrication of near-infrared LEDs [17, 24] and near-infrared lasers applied in sensing and probing.

**Fig. 3:**
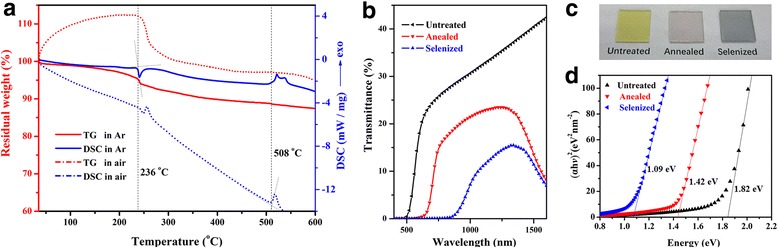
Photoelectric properties of Sb_2_S_3_ QD thin films. **a** TG and DSC profiles of dried Sb_2_S_3_ QDs tested in Ar and air. **b** Transmittance spectrum, **c** a photograph, and **d** band gap analysis of pre- and post-annealed Sb_2_S_3_ QD thin films

To further study the applications of Sb_2_S_3_ QDs in semiconductor processing, Sb_2_S_3_ films were prepared by self-assembly growth on glass from a 5 mg/ml QDs-isopropanol dispersion. Before anneal treatment, thermogravimetric analysis was employed for the stability test. According to TG and DSC profiles for the vacuum-dried QDs shown in Fig. 3a, Sb_2_S_3_ QDs have an approximately 12% weight increment beginning from room temperature, indicating these have a high activity and probably been partly oxidized or surface-adhered. Sb_2_S_3_ QDs exhibit a relative stability in argon at room temperature and then show the first obvious weight loss followed by the excess S removal started at 236 °C. The melting point of Sb_2_S_3_ QDs was measured to be 508 °C, which is remarkably lower than that of crystalline Sb_2_S_3_ powder (550 °C, Sigma Aldrich). We noticed there was a gradual slow weight loss at the whole test temperature range accompanied by S constituent loss. Sb_2_S_3_ QD films anneal treatment in Ar and Se vapor was subsequently studied. Optical transmission spectra for untreated, annealed, and selenized films are shown in Fig. 3b, and the photograph of the three samples is shown in Fig. 3c. For the annealed and selenized samples, both of them were treated at 250 °C for 5 min. The absorbing edges of the annealed and selenized samples were shifted from 500 nm to 650 and 850 nm, respectively. Because both Sb_2_S_3_ and Sb_2_Se_3_ are direct band gap semiconductor [24, 25], the average band gap could be calculated by the formula:

3 $$ \upalpha =\left(A/\mathrm{h}\upnu \right)\times {\left(\mathrm{h}\upnu -\mathrm{Eg}\right)}^{1/ 2} $$

where *A* is a constant, *h* is Planck’s constant, and *ν* is the frequency of the incident photon. We fitted the linear zone by plotting (*αhv*)^2^ versus (*hv*) and calculated the average *E*_*g*_ as shown in Fig. 3. As we see, the *E*_*g*_ of untreated sample was 1.82 eV and decreased to 1.42 eV after 5 min annealing at 250 °C. The variation of *E*_*g*_ indicates the crystallinity of Sb_2_S_3_ has been improved with an order-disorder transformation accompanied by the removal of excessive S element [26]. For the selenized sample, the *E*_*g*_ decreased to a minimum of 1.09 eV, which is very close to crystalline silicon. Quantitative elemental EDS analysis revealed that Sb_2_S_3_ was transformed to Sb_2_(S_1−*x*_Se_*x*_)_3_ and finally to Sb_2_Se_3_ after the most of sulfur have been replaced by selenium [7, 9]. Because the selenylation is lower than 250 °C, we believe it was beneficial for the manufacturing and performance improving of flexible devices. As we know, the optimum band gap for solar cell absorber was 1.45 eV. Thus, the annealed and selenized Sb_2_S_3_ QD films are good candidates for photovoltaic absorber materials.

## Conclusions

A novel way to synthesize water-soluble Sb_2_S_3_ QDs was developed by hot injection using CTAB and SDS mixture as anionic-cationic surfactant, DEA as stabilizer, and EDTA as dispersant. The synthesis process is easy to operate and repeatable. All the reagents and additives are nontoxic, odorless, and inexpensive. Sb_2_S_3_ QDs have an intensive PL emission at 880 nm and a high transmittance at a near-infrared region, indicating it has good prospects in the fabrication of near-infrared LEDs and near-infrared lasers. Sb_2_S_3_ QDs show a good monodispersity and processibility, which can be deposited to form Sb_2_S_3_ films. The *E*_*g*_ of Sb_2_S_3_ QD films could be turned to 1.42 and 1.09 eV after annealing treatment in Ar or Se vapor at lower than 250 °C, demonstrating their good prospects in photovoltaic application.

## Abbreviations

CBD: Chemical solution deposition
CTAB: Hexadecyltrimethylammonium bromide
DEA: Alkanol amide
DSC: Differential scanning calorimeter
EDS: Energy-dispersive spectrometer
EDTA: Ethylenediaminetetraacetic acid
HRTEM: High-resolution transmission electron microscopy
LED: Light-emitting diode
OLED: Organic light-emitting diode
PL: Photoluminescence
QD: Quantum dot
QSE: Quantum size effect
SAED: Selected-area electron diffraction
SDS: Sodium dodecyl sulfate
STA: Simultaneous thermal analyzer
TAA: Thioacetamide
TGA: Thermogravimetric analysis
XRD: X-ray diffraction
